# A QbD Approach for Evaluating the Effect of Selective Laser Sintering Parameters on Printability and Properties of Solid Oral Forms

**DOI:** 10.3390/pharmaceutics13101701

**Published:** 2021-10-15

**Authors:** Yanis A. Gueche, Noelia M. Sanchez-Ballester, Bernard Bataille, Adrien Aubert, Jean-Christophe Rossi, Ian Soulairol

**Affiliations:** 1 ICGM, CNRS, ENSCM, University Montpellier, 34000 Montpellier, France; yanis-abdelhamid.gueche@etu.umontpellier.fr (Y.A.G.); noelia.sanchez-ballester@umontpellier.fr (N.M.S.-B.); bernard.bataille@umontpellier.fr (B.B.); adrien.aubert@umontpellier.fr (A.A.); 2 Department of Pharmacy, Nîmes University Hospital, 30900 Nimes, France; 3 IBMM, University Montpellier, CNRS, ENSCM, 34000 Montpellier, France; jean-christophe.rossi@umontpellier.fr

**Keywords:** 3D printing, selective laser sintering, process parameters, solid oral forms, copovidone, paracetamol, printability, Quality by Design, design of experiments

## Abstract

The aim of this work was to investigate the effect of process parameters on the printability of a formulation containing copovidone and paracetamol, and on the properties of solid oral forms 3D-printed through selective laser sintering. Firstly, the influence of the heating temperature was evaluated individually, and it was revealed that this parameter was critical for printability, as a sufficiently high temperature (100 °C) is necessary to avoid curling. Secondly, the effects of laser power, scan speed, and layer thickness were determined using a Box–Behnken design. The measured responses, printing yield, height, weight, hardness, disintegration time, and percentage of drug release at 10 min showed the following ranges of values: 55.6–100%, 2.92–3.96 mm, 98.2–187.2 mg, 9.2–83.4 N, 9.7–997.7 s, and 25.8–99.9%, respectively. Analysis of variance (ANOVA) proved that the generated quadratic models and the effect of the three–process parameters were significant (*p* < 0.05). Yield improved at high laser power, low scan speed, and increased layer thickness. Height was proportional to laser power, and inversely proportional to scan speed and layer thickness. Variations in the other responses were related to the porosity of the SOFs, which were dependent on the value of energy density. Low laser power, fast scan speed, and high layer thickness values favored a lower energy density, resulting in low weight and hardness, rapid disintegration, and a high percentage of drug release at 10 min. Finally, an optimization was performed, and an additional experiment validated the model. In conclusion, by applying a Quality by Design approach, this study demonstrates that process parameters are critical for printability, but also offer a way to personalize the properties of the SOFs.

## 1. Introduction

Three-dimensional printing, also known as additive manufacturing, is a versatile technology that enables the production of devices with various applications [1]. This state-of-the-art technology occupies an important place in society, and has proved to be helpful and efficient on several occasions [2]. For example, during the 2019 coronavirus disease pandemic (COVID-19), 3D printing provided a quick and easy solution to the shortage of protective equipment (such as masks and face shields) and medical instruments (ventilator valves) [3,4]. In healthcare, 3D printing is not only applied in dentistry, medical devices, and organ and tissue transplantation, but also in the fabrication of solid oral forms, usually called “printlets” [5].

This kind of printing allows for the production of an object in a layer-by-layer fashion, according to its pre-established design produced with computer-aided design (CAD) software [6]. In this way, various geometries can be translated into solid oral forms (SOFs). This flexibility could help the development of personalized medicine by providing tailored medicines on-demand [7]. Moreover, printing parameters also participate in the modulation of the inner structure, thereby adapting the properties of the SOFs to patients’ needs. For example, in extrusion-based 3D printing techniques, such as fused deposition modeling (FDM) and semi-solid extrusion (SSE), the infill density is a critical parameter that regulates the percentage of pores in the printlet, which could accelerate or, contrarily, slow down drug release, depending on its value [8,9]. In powder bed fusion (PBF) techniques, the process parameters also influence the structure and the properties of the printlets [10]. Selective laser sintering (SLS) is a PBF technique that uses a laser beam to fuse the powder particles together to form a solid object [11]. This technology has been explored for the production of drug delivery devices (DDDs) [12,13,14], and, more recently, for the printing of SOFs [15,16,17]. Its main outcome for pharmaceutical applications lies in its ability to generate porous structures [18], such as porous scaffolds for bone regeneration [19] and porous printlets for oral disintegration [20]. Porosity can be obtained either by creating voids in the original design, or by varying the printing parameters. For instance, the laser energy density (ED) is the amount of energy transmitted by the laser per unit volume of powder and is a function of four processing parameters as shown in the empirical equation:

(1) $$ED\;\left(J/mm^{3}\right)=\frac{LP}{SS\times HS\times LT}$$

where *LP, SS, HS and LT* are, respectively, laser power, scan speed, hatch spacing, and layer thickness. This relation demonstrates not only that the degree of sintering is proportional to the laser power, but also that it increases with slow scan speed, low hatch spacing, and reduced layer thickness. For example, Fina et al. developed highly porous and fast disintegrating printlets by increasing the laser scan speed [21]. More recently, hatch spacing was explored as a way to modulate porosity and control drug release from high dosage printlets [22]. Laser power and layer thickness remain unexplored in the context of SOF’s manufacturing by SLS.

However, the process parameters not only influence the properties of printlets, but also their ability to be printed [23], known as *printability*. As suggested by Chatham et al., the critical parameters for sintering a defect-free object may differ from those needed to obtain an object with specified properties [24]. For instance, lowering the heating temperature can improve the porosity of printed devices, as demonstrated by Low et al. [25]. On the other hand, a sufficiently high temperature is critical for the process, as it reduces the thermal gradient between the unsintered region and the sintered powder, and prevents curling of the sintered layers, allowing for successful printing [24,26]. Therefore, printability of pharmaceutical materials as a function of printing conditions should be studied for the preparation of SOFs by SLS.

A systematic approach to study the influence of process parameters is the design of experiments (DoE). This analytical tool has earned incredible consideration in pharmaceutical research since the introduction of the concept “Quality by Design” (QbD) by the Food and Drug Administration (FDA) in pharmaceutical development [27]. In QbD, a thorough understanding of the product and the process is paramount to ensure the quality in the final product. This mainly involves identifying the quality target product profile and its critical quality attributes (CQAs). After that, the critical process parameters (CPPs) that may affect the CQAs of the product are determined with the help of a DoE. As a result, an operational design space is generated in which the product quality is guaranteed [28]. DoE have been already applied on 3D printing of medicines. For example, Korte et al. used a full factorial design to assess the effect of the hot melt extrusion (HME) parameters on producing a printable filament with an accurate diameter [29]. 

Design of experiments was also employed in SLS, specifically to explore process and formulation variables that may affect the quality of the printlets. Barakh Ali et al. evaluated the influence of the independent variables (chamber temperature, laser scan speed, and lactose monohydrate concentration) on the dependent variables (weight, hardness, disintegration time, and fraction of drug dissolved in 15 min) [30]. This was followed by the work of Mohamed et al., focusing on formulation optimization for the SLS of printlets containing clindamycin palmitate hydrochloride [31]. 

In this context, the aim of this work was to study the influence of selective laser sintering parameters on both printability and properties of the printed solid oral forms prepared with copovidone and paracetamol, utilizing a Quality by Design approach. First, the impact of the heating temperature was evaluated individually, and an optimal temperature was chosen based on the processability of the powder mixture. Secondly, the effects of laser power, scan speed, and layer thickness on the quality of printed SOFs were assessed using a design of experiments. Finally, optimization and a confirmation test were performed to determine the optimal setting and validate the model.

## 2. Materials and Methods

### 2.1. Materials

Kollidon^®^ VA64 (Copovidone) (KVA64) was donated by BASF (Ludwigshafen, Germany), whilst paracetamol (PAR) was provided by Sequens (France). Hydrochloric acid (37%) for the preparation of the dissolution medium was purchased from Carlo Erba Reagents (Milano, Italy). 

For this work, a formulation of 90% of KVA64 and 10% of PAR was chosen as the powder feedstock. Mixing was conducted on a 3D shaker mixer Turbula^®^ T2F (WAB, Muttenz, Swizterland) at a speed of 49 rpm for 10 min.

### 2.2. Printing of Solid Oral Forms 

Printing was conducted on a 3D SLS printer Sharebot^®^ SnowWhite (Sharebot, Nibionno, Italy) equipped with CO_2_ laser (λ = 10.6 µm). The design of the solid oral form consisted in a cylinder (10 mm diameter and 2.4 mm height), and was realized with the online CAD software OnShape^®^ (Onshape, Boston, MA, USA), then exported as a STL file. The latter was converted to a G-code with the open-source software Slic3r^®^ 1.2.9, before being transferred to the SLS printer. 

For each printing run, a mass of 200 g from the formulation was loaded in the reservoir tanks and the building platform, and a batch of 36 SOFs was launched for printing. The process started with the heating of the powder using infrared lamps (230 W). Afterwards, the CO_2_ laser scanned and consolidated the successive powder layers deposited by the recoater blade. At the end of printing, the printed SOFs were retrieved from the building platform, and their powder excess was brushed away.

The four controlled printing parameters in this study were heating temperature (*HT*), laser power (*LP*), scan speed (*SS*), and layer thickness (*LT*). *HT* (°C) corresponded to the temperature of the powder bed, as the temperature mode was set to “powder temperature”. *LP* was expressed as percentage (%) of the maximum laser power (14 W). The unity used for *SS* was pps (points per seconds), and 1 pps corresponds approximately to 0.05 mm/s. While these aforementioned parameters were set on the printer screen, *LT* (µm) was entered in Slic3r^®^.

Prior to the design of the experiments, preliminary tests were carried out to determine the optimal temperature for printing, which corresponded to the temperature at which the printing yield was maximum (100%).

(2) $$Yield=\frac{number\;of\;printed\;SOFs\;with\;no\;defects}{number\;of\;SOFs\;launched\;for\;printing}\times 100\%$$

For this purpose, five different temperatures were assessed: 90, 95, 100, 105, and 110 °C. The other printing parameters were set as a constant: *LP* = 30%, *SS* = 35,000 pps and *LT* = 100 µm. 

### 2.3. Design of Experiments

**Figure 1:**
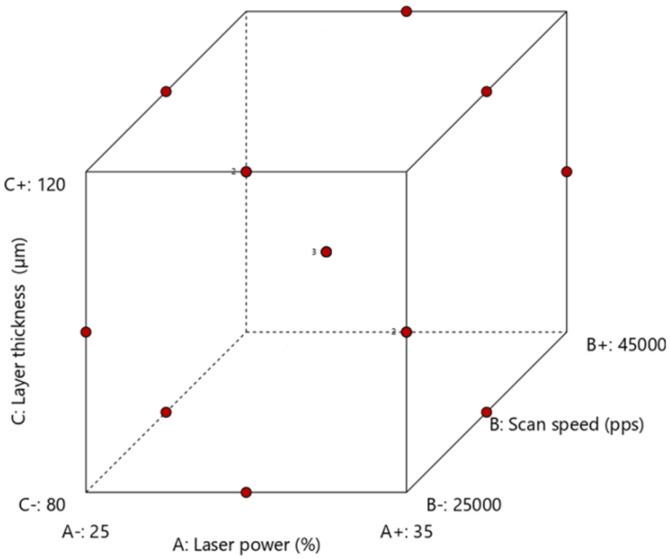
Box–Behnken design with the design points.

In order to study the influence of process parameters, a three-level, three-factor, Box–Behnken design (Figure 1) was generated using the software Design-Expert^®^ 13 (Stat-Ease Inc, Minneapolis, MN, USA). The levels of the different factors were selected based on preliminary trials. The evaluated factors or independent variables were laser power *LP* (A, %), scan speed *SS* (B, pps), and layer thickness (C, µm). The measured responses or dependent variables were yield (Y_1_, %), height (Y_2_, mm), weight (Y_3_, mg), hardness (Y_4_, N), disintegration time (Y_5_, s), and percentage of drug release at 10 min (Y_6_, %). In total, 15 experimental runs were produced by the software, including three central points used to measure the reproducibility of the process. Table 1 summarizes the printing parameters for each run. 

Each response was fitted with a quadratic model as following:

(3) $$Y=O_{0}+O_{A}\;A+O_{B}\;B+O_{C}\;C+O_{AB}\;AB+O_{AC}\;AC+O_{BC}\;BC+O_{AA}\;A^{2}+O_{BB}\;B^{2}+O_{CC}\;C^{2}$$

*O_0_* is the intercept; *A*, *B* and *C* are the linear terms; *AB*, *AC* and *BC* are the 2 factors interactions terms; *A^2^*, *B^2^* and *C^2^* are the quadratic terms, *O_A_*, *O_B_*, *O_C_*, *O_AB_*, *O_AC_*, *O_BC_*, *O_AA_*, *O_BB_* and *O_CC_* are the estimated coefficients of *A*, *B*, *C*, *AB*, *AC*, *BC*, *A^2^*, *B^2^* and *C^2^*, respectively. 

Analysis of variance (ANOVA) was performed to evaluate the significance of the model and its different terms (α = 0.05). 

At the end, an additional experiment was conducted with the optimized printing settings, using the Design Expert tool “numerical optimization” in order to validate the model. 

### 2.4. Characterization of the Printed SOFs

#### 2.4.1. Physical Characterization (Weight, Dimensions, Hardness, Disintegration Time) 

The weight of the SOFs was determined using precision electronic balance Adventurer^®^ (OHAUS, Parsippany, NJ, USA). Height and hardness were measured using a Sotax Multitest 50FT (Sotax AG, Aesch, Switzerland). Measurements were carried out on 5 tablets per printing run, and results were expressed as the mean value ± standard deviation.

Disintegration tests were performed on a disintegration apparatus (Sotax DT50, Sotax AG, Switzerland) with distilled water (800 mL) at 37 °C, according to the European Pharmacopeia guidelines [32]. For each printing run, six SOFs were tested simultaneously. The disintegration time was reported as the mean value ± standard deviation.

#### 2.4.2. Fourier Transform Infrared Spectroscopy (FTIR) Analysis 

FTIR spectrums of KVA64, paracetamol, the physical mixture, and the printed SOFs (Run 14) were recorded using the infrared spectrophotometer Vector 22 FTIR (Bruker, Billerica, MA, USA). Data was collected from 4000 to 400 cm^−1^ at room temperature (approximately 25° C), and 32 scans were averaged at a resolution of 2 cm^−1^. Samples of 100 mg were prepared by blending 10 mg (polymer, physical mixture, crushed SOF) or 1 mg (drug) with Q.S. (Quantum satis) of anhydrous potassium bromide (previously dried in the oven at 100 °C for 30 min), and compressing the mixture to form a disk. The FTIR spectrums were treated using the infrared software OPUS 6.5.

#### 2.4.3. X-ray Powder Diffraction (XRPD) Analysis

The solid states of the polymer, drug, physical mixture, and printed SOFs (Runs 1, 3, 7, 11, 14) were characterized using a Bruker D8 Advance diffractometer (Bruker, USA) and the monochromatic Cu Kα1 radiation (λα = 1.5406 Å, 40 kV, and 40 mA). For the case of the physical mixture and the printed SOFs, the analyzed samples were disks prepared by compressing 100 mg of powder in order to compare the intensity of the crystalline peaks at the same weight. The angular range of data recording was 2–70° 2Ɵ, with a stepwise size of 0.02° and a speed of 0.1 s counting time per step, using LINXEYE detector 1D.

#### 2.4.4. Scanning Electron Microscopy (SEM)

Porosity of the printed SOFs (Runs 1, 3, 7, 11, 14) was assessed through the visualization of the vertical sections by scanning electron microscopy (SEM). Samples were prepared by cutting thin vertical layers of SOFs with a blade, then were fixed on a support with adhesive tape. Images were taken with the scanning electron microscope (Hitachi 4800 S) after platinum sputtering under vacuum. 

### 2.5. Drug Content in the Printed SOFs

For each printing run, three individual SOFs were dissolved in 100 mL of distilled water. Samples of the solutions were then diluted, and the drug concentration was determined through ultra-high performance liquid chromatography (UHPLC) using a UHPLC-DAD system. It consisted of a Thermo Scientific™ Dionex™ UltiMate™ 3000 BioRS equipped with a WPS-3000TBRS autosampler, and a TCC-3000RS column compartment set at 35 °C (Thermofisher Scientific, Waltham, MA, USA). The system was operated using Chromeleon 7 software. An Accucore C18 column (2.6 µm, 100 × 2.1 mm) combined with a security guard ultra-cartridge (Phenomenex Inc., Torrance, CA, USA) was used. An isocratic binary solvent system was utilized, consisting of water/formic acid (0.1%, *v*/*v*) as solvent A, and acetonitrile/formic acid (0.1%, *v*/*v*) as solvent B (90%A, 10%B). The flow rate of the mobile phase was 1.5 mL/minute, and the injection volume was 50 μL. Quantitative analysis of paracetamol in the SOFs was carried out using an external standard method. The calibration curve was constructed using 5 different standard levels in the concentration range 1–20 mg/L. The peak of paracetamol was monitored at 244 nm. 

### 2.6. In-Vitro Dissolution Study of the Printed SOFs

A dissolution test was carried out with a Pharma Test DT70 dissolution tester (Hainburg, Germany) using a paddle apparatus (European Pharmacopeia) [33]. For each printing run, three SOFs were randomly selected and individually placed in the dissolution vessels, each containing 900 mL of 0.1 M HCl (sink condition), and stirred at 50 rpm and 37 ± 0.5 °C. Samples were analyzed automatically every 5 min using a continuous flow-through system attached to an 8 cell UV/Vis spectrophotometer Specord 250 (Analytik Jena, Jena, Germany) at the wavelength of 244 nm. Results were expressed as mean values with standard deviation.

### 2.7. Drug Release Kinetics Models

The study of drug release kinetics was performed with the KinetDS 3.0 software. The dissolution data for the SOFs printed at different runs were fitted to several mathematic models, including the zero-order, Korsmeyer–Peppas, Weibull, Higuchi, Michaelis–Menten, and Hill models. In order to evaluate the accuracy of the individual models, the root mean square error (RMSE) was calculated as following:

(4) $$RMSE=\sqrt{\frac{\sum_{i=1}^{n}{\left(y_{i}-{\hat{y}}_{i}\right)}^{2}}{n}}$$

The RMSE describes the differences between the measured values *y_i_* and the model-predicted values *ŷ_i_*; thus, a low value of RMSE indicates that the model is accurate. The number of timepoints is indicated by *n*. 

## 3. Results and Discussion

### 3.1. Effect of Heating Temperature on Powder Printability and SOFs Properties

**Figure 2:**
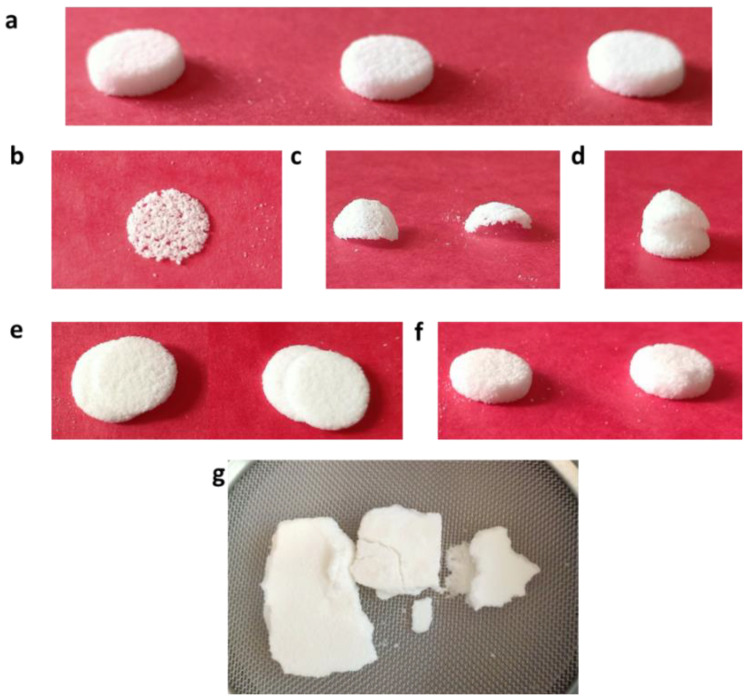
Printing defects in SLS: (**a**) SOFs fully printed with no defects; (**b**) sintered single layer; (**c**) curled single layer; (**d**) SOF with curled upper layers; (**e**) SOF with shifted layers; (**f**) SOFs surrounded by solidified powder; and (**g**) powder cake.

In this first part of the study, the effect of heating temperature (*HT*) was evaluated on both the processability and properties of SOFs. Table 2 displays the printing yield at five different temperatures. The optimum *HT* at which all the SOFs were printed with no defects (Figure 2a) was 100 °C. For amorphous polymers, such as copovidone, the heating temperature was set above the glass transition temperature (Tg) [20]. As previously demonstrated [34], the Tg of KVA64 is around 103 °C, and is reduced when paracetamol is introduced (Tg = 80.3 °C). This explains the lower optimal *HT* for the mixture, compared to pure KVA64 (110 °C).

When *HT* was lowered, printability was affected, and the printing yield decreased drastically (Table 2). As suggested by Goodridge et al. [35], before and during laser sintering, the powder must be heated at a sufficiently high temperature for three main reasons: (i) to minimize the required sintering energy provided by the laser; (ii) to limit the thermal expansion of the powder due to the laser; and, most importantly, (iii) to avoid a thermal shock between the consolidated particles and the surrounding powder that could result in a curling of the sintered layers. At 95 and 90 °C, the produced SOFs exhibited many printing defects (Figure 2) that were all related to shrinkage and curling of the sintered powder layers. It is important to note that, in the case of amorphous polymers, shrinkage is not influenced by crystallization, but is favored by a low powder compactness and high interparticular porosity [36], which has already been demonstrated for copovidone [34]. When a new layer of powder is deposited, the curled layers can be dragged by the recoater blade, and discharged into the recycling bins where they are usually found. Depending on the importance of the thermal gradient, curling can be observed with the naked eye (Figure 2c), or not (Figure 2b). It can also occur at the late stage of sintering, resulting in curling of the upper layers only (Figure 2d). When a curled layer is just slightly displaced by the recoater blade from its initial position and then binds to the subsequent sintered layer, shifting may occur (Figure 2e). 

Additionally, when *HT* was increased, the printing yield declined, but less significantly (Table 2). Augmenting the heating temperature could lead to an excessive consolidation of the powder particles, including those that were not exposed to the laser [37], resulting in a phenomenon known as “powder cake” (Figure 2g). Consequently, the retrieving and brushing of the SOFs would be problematic due to the consolidated particles around the printed part (Figure 2f). At higher temperatures, powder cake could even prevent the recoater blade from depositing a new layer of powder [37]. 

Furthermore, it is also possible to predict the curling phenomenon before retrieving the produced SOFs, by observing the aspect of the powder bed during and at the end of the sintering process. If curling occurs, the powder bed will be cracked, else it will be flat (Figure S1, Supplementary Materials).

Nevertheless, the properties of the SOFs printed at the three different temperatures (95, 100, 105 °C) were evaluated. Table 3 shows that, in general, the height, weight, hardness, disintegration, and percentage of drug release at 10 min did not change significantly when the temperature was changed from 95 to 100 °C. However, at 105 °C, the weight, height, and disintegration time increased, whereas the fraction of dissolved drug at 10 min (Figure S2, Supplementary Materials) decreased by approximately 6%. This could be explained by the more important consolidation of the powder due to the higher temperature [38], which results in denser SOFs that disintegrate and dissolve more slowly. As for the increased height, it could be related to the more important reduction of the thermal gradient at 105 °C, which prevents the SOFs from shrinkage [39]. Barakh Ali et al. studied the effect of heating temperature, and demonstrated its significant influence on the quality of printlets by affecting the degree of powder consolidation [30]. However, the increment in temperature was higher (10 °C) than in this study (5 °C).

Overall, heating temperature was found to be a determinant for the printability of the powder feedstock, since a decrease or increase of 5 °C affected the yield, limiting the processability at different temperatures. However, the properties of the printed SOFs were not significantly influenced by these small temperature variations. 

### 3.2. Effect of Process Parameters on Powder Printability and SOFs Properties

Box–Behnken design is a three-levels design, frequently used for fitting response surfaces [30,31]. Opposed to a 3^3^ full factorial design, it does not include design points at the vertices of the cube where all factors are at the upper or lower levels (Figure 1). This is advantageous when these combinations are impossible due processing constraints. It is important to highlight that, despite the low number of required runs, the design is efficient for process optimization [40]. The values of the measured responses for each run are presented in Table 4. 

#### 3.2.1. Analysis of Variance (ANOVA)

ANOVA was carried out to confirm the significance of the model and the different terms for each response (Table 5).

For each response, a quadratic model was used, as it fits the data appropriately. As shown in Table 5, the large F-values of the models imply that the difference between the means is due to real effects, and not to error. Model *p*-values were smaller than α = 0.05, indicating that all models are significant. Model terms with *p*-values < 0.05 are significant, whereas values greater than 0.10 indicate insignificance. For instance, the three independent variables (laser power, scan speed, and layer thickness) were significant terms in the models of the six responses. However, the significance of interaction and quadratic terms varied, depending on the modelized response. Insignificant terms could be excluded to obtain an improved reduced quadratic model, but this was not performed since it would not allow the study of the interaction between the independent terms. Lack of fit evaluates the fitting efficiency of the model, and is measured from the center points (Runs 10, 12 and 15). For each of the values of height, weight, hardness, disintegration time, and percentage of drug release at 10 min, the lack of fit was not significant (*p*-value > 0.05), confirming that the model fits adequately. As for the yield, there was not lack of fit since the triplicates presented the same response (100%). The predicted R^2^ was in reasonable agreement with the adjusted R^2^ for the four dependent variables: height, weight, disintegration time, and drug release at 10 min. This was not the case for yield and hardness, as the difference between adjusted R^2^ and predicted R^2^ was greater than 0.2. This was due to the inclusion of non-significant terms in their respective models. Adequate precision measures the signal-to-noise ratio, and since the values for all six models were greater than 4, this implied an adequate signal. 

#### 3.2.2. Model Diagnostics

The quadratic models can be represented by equations expressing the empirical relationship between the properties of the printed SOFs (responses Y) and the process parameters (factors A, B, C), as shown in Table 6. The significant terms are indicated in bold. Depending on the sign of the coefficient, the term would have a positive or a negative effect on the response. 

In order to improve the model, a response transform can be recommended by the diagnostic tools in Design-Expert^®^, such as the Box-Cox plot. Thus, instead of an equation of the type y = *f* (x), the model will be expressed by an equation of the type *f* (y) = *f* (x), as is the case for disintegration time and percentage of drug release at 10 min. For disintegration time, the difference between the mean values was important (ranging from 9.7 to 997.7 s); therefore, an inverse square root transform was conducted $\frac{1}{\sqrt{Y_{5}-0.5}}$ (Table 6). However, for the percentage of drug release at 10 min, the difference was not important, as the values of 10 runs were all fluctuating between 90 and 100%. Hence, a power transform of *Y*_6_^2.21^ was used (Table 6). 

The adequacy of the generated models was verified with the residuals vs. run plots (Figure S3, Supplementary Materials), and the predicted vs. actual values plots (Figure S4, Supplementary Materials). The residual is the difference between the actual and predicted values for each design point. In the residual vs. run plot for each response (Figure S3), the values are randomly scattered around the x-axis, indicating that the errors are normally distributed. Furthermore, Figure S4 shows a good correlation between the actual and predicted values for the six dependent variables. Based on the values of the correlation coefficient (R^2^), the responses can be ranked in an increasing order of predictivity as follows: weight > percentage of drug release at 10 min > disintegration time > height > hardness > yield. 

#### 3.2.3. Effect of Process Parameters on Printability

**Figure 3:**
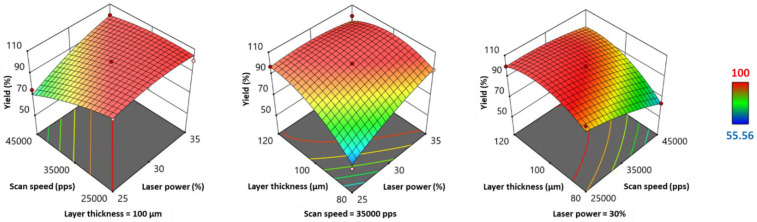
3D response surface plots for printing yield.

Yield was used to quantify printability, and a low value indicated a high number of SOFs printed with defects caused by shrinkage and subsequent curling. Based on the values of the coefficients associated with the individual factors (Table 6), the yield was significantly and positively influenced by laser power (*LP*) and layer thickness (*LT*), while scan speed (*SS*) presented a significant negative effect on the response. Figure 3 represents the 3D response surface plots for the yield. These plots help to evaluate the interaction of two parameters while a third one is set as a constant. It should be noted that a decrease in *LP* affects the yield more at high *SS* (45000 pps) than at low *SS* (25000 pps), demonstrating a negative influence of the interaction LP*SS on the yield. In addition, low *LT* and high SS show a synergistic negative effect on printability. Figure 3 also shows an important decrease in the yield when low *LP* and low *LT* are combined. However, LP*LT was the only significant interaction, as shown in Table 6. The square of *LT* also showed a significant negative effect on the yield.

Variations of printing yield can be explained by the effect of process parameters on curling. Similar to these observations, Wang et al. [39] reported an increase in shrinkage with increasing scan speed, but a decrease with increasing layer thickness, laser power, and powder bed temperature. However, during preliminary tests, it was observed that increasing *LP* and/or decreasing *SS* excessively can also affect printability, and the resulting printed SOFs were curled and yellow, suggesting degradation. These variations caused an increase in energy density and maximize the thermal gradient between the sintered region and the unsintered powder, inducing curling [38].

**Figure 4:**
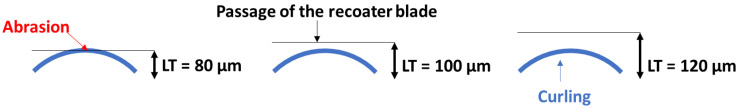
Schema depicting the abrasion of curled layers at *LT* = 80 µm.

Furthermore, the influence of layer thickness on printability was not only reliant on shrinkage. In order to understand this, it is elementary to define layer thickness. This parameter as input in the software (theoretical value) corresponds to the height of the powder layer deposited on the powder bed, and is different from the height of the sintered layer (experimental value), which depends on the material used and the parameters applied. According to the literature, it should be higher than the particle size (D90: diameter where 90% of the particles distribution has a smaller particle size) [41], and lower than the laser penetration characterized by the optical penetration depth (OPD) [42]. This implies that the powder layer must be thick enough so that the particles are not abraded when the blade comes to deposit a new layer, but also thin enough so that the laser can penetrate and bind two subsequent layers. In this study, a decrease in layer thickness *(LT* = 80 µm) reduced the printing yield not only by curling but also by abrasion of the sintered layers, especially when other favorable factors of curling, such as low laser power (Run 9) or high scan speed (Run 13) (Table 4), were present. Figure 4 schematizes the abrasion and the dragging of curled layers by the recoater blade when a new powder layer is deposited.

On the other hand, increasing the layer thickness value to any limit will not improve yield. During preliminary trials, when layer thickness was set to 160 µm (≈D90) [34], the printed SOFs were very fragile, and crumbled when handled. This could be due to an OPD lower than the layer thickness, and therefore insufficient to bind two successive layers. Thus, a maximum value of 120 µm was selected to evaluate the influence of the layer thickness.

It is interesting to note that the printing yield was not a binary response (0%: failed printing for all the programmed SOFs, 100%: successful printing for all the programmed SOFs). For instance, in non-optimal process conditions (in terms of printability), more than 50% the SOFs were still printed with no defects. This demonstrates heterogenous sinterability in the printing bed. An even temperature distribution in commercial machines can exist, with the peripheral areas being cooler than the center of the printing bed [35]. However, the other process parameters may also have a different effect depending on the position of SOF in the printing bed. 

Printability is an important aspect in SLS that is often neglected in other studies. It provides information on how raw material and printers can be optimized to improve processing. Esthetic defects that may be present in SOFs (Figure 2) could weaken their acceptability by patients, and impact treatment compliance [43], which would go against the paradigm defended by 3D printing “personalized medicine”. Therefore, printability seems to be a necessary condition prior to the modulation of the SOF’s properties. 

#### 3.2.4. Effect of Process Parameters on Height

**Figure 5:**
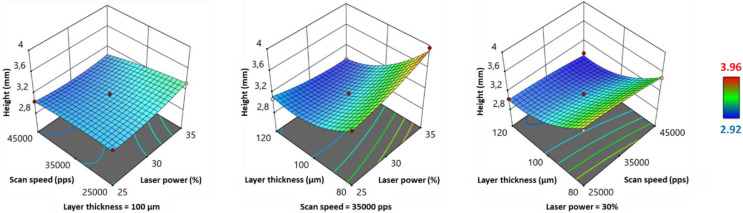
3D response surface plots for height.

According to the model Equation (Table 6), the laser power (*LP*) had a significant positive effect on the height of the printed SOFs, and the layer thickness (*LT*) showed a significant negative effect. On the other hand, scan speed (*SS*) exhibited a significant but weak positive influence (low value of the coefficient). Furthermore, the LP*SS and LP*LT interactions had a negative effect on the height, which was also visible in the 3D response surface plots (Figure 5), as the maximum values were situated in the right corner. The SS*LT interaction presented a minimal influence (coefficient = 9 × 10^−8^), as the height increased incrementally in layer thickness, independently of the value of scan speed (Figure 5). In terms of significance (Table 6), only the LP*LT interaction was significant (*p* < 0.05). Moreover, the quadratic term *LT^2^* demonstrated a significant positive effect on the height (Table 6).

Height appears to be mainly influenced by two physical phenomena: laser penetrance, and shrinkage. Laser penetrance is more profound at high energy densities [42], resulting in an excessive growth along the z-axis, called *bonus z* [35]. This explains the increase in height of SOFs when the laser power is increased, or the scan speed and layer thickness are reduced. On the other hand, shrinkage can occur when the polymer cools down at the end of printing, or even during the delay time while a new layer is deposited on top of the previously sintered layer. The importance of shrinkage at low laser power and fast scan speed [39] could explain the smaller dimensions of the printed SOFs under these conditions. As for the considerable decrease in height when switching the layer thickness from 80 to 120 µm (Figure 5), this can be explained by the reduction of the number of sintered layers, thus the number of deposited particles. The number of sintered layers depends on the layer thickness input in the slicer: 30, 24, or 20 layers, depending on whether *LT* is 80, 100, or 120 µm. Some particles are larger than the layer thickness (D90 > 80 µm), and stacking them in 30 layers, compared to 20 layers, could widely enlarge the tablets. Although height values varied from 2.92 to 3.96 mm (Table 4), all of them were greater than the design input value (2.40 mm). The height is directly related to the thickness of the sintered layer, which depends on the material and process parameters. For example, it has previously been demonstrated that powder with low packing density rises the OPD [44], and this can constitute an explanation for the relatively higher SOFs obtained with copovidone [34]. For all printing runs, the average sintered layer thickness (ASLT) was calculated as follows:

(5) $$ASLT=\frac{height\;of\;the\;sintered\;SOF}{number\;of\;sintered\;layers}$$

Table S1 (Supplementary Materials) shows that, for all printing runs, the average sintered layer thickness exceeded the corresponding input value. The ASLT increased with the *LT* value: ASLT = 116.3–131.9 µm for *LT* = 80 µm vs. ASLT = 146.1–153.6 µm for *LT* = 120 µm. The variation of ASLT between different runs, despite a constant *LT*, also proves that other process parameters such as *LP* and *SS* have an influence by affecting OPD and shrinkage. A reduction of *LT* could help to achieve the desired height for the designed SOF, but it would affect printability greatly, as explained above. Therefore, the only remaining option to obtain SOFs with a target height appears to reduce the value in the design before printing.

#### 3.2.5. Effect of Process Parameters on Weight, Hardness, Disintegration Time and Percentage of Drug Release at 10 min

**Figure 6:**
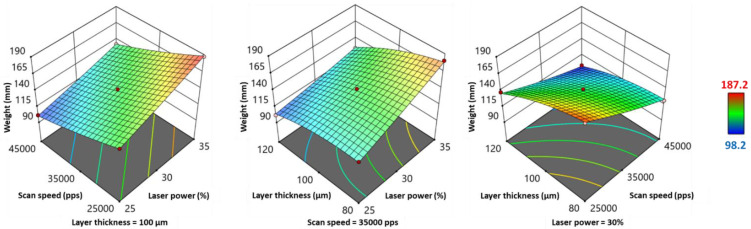
3D response surface plots for weight.

**Figure 7:**
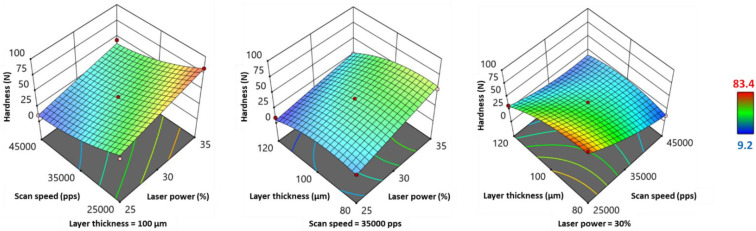
3D response surface plots for hardness.

The weight and hardness of the printed SOFs presented similar variations based on their quadratic model equations (Table 6) and 3D response surface plots (Figure 6 and Figure 7). Scan speed (*SS*) demonstrated a significant negative effect on both responses, whereas laser power (*LP*) and layer thickness (*LT*) exhibited a significant positive effect (Table 6). Regarding the factors’ interactions, LP*SS and LP*LT displayed a negative influence on weight and hardness, as their values (Figure 6 and Figure 7) were the highest at the right hand corner (high *LP* and low SS, high *LP* and low *LT*). SS*LT showed a positive influence on both responses, and the highest values were situated at the bottom corner (low *SS* and low *LT*). Regarding weight, LP*LT and SS*LT had a significant effect, whereas for hardness, only the effect of SS*LT was significant (*p* < 0.05) (Table 6). As for quadratic terms, *LT^2^* exhibited a significant negative effect on both responses, and *SS^2^* had a significant positive effect only on hardness.

**Figure 8:**
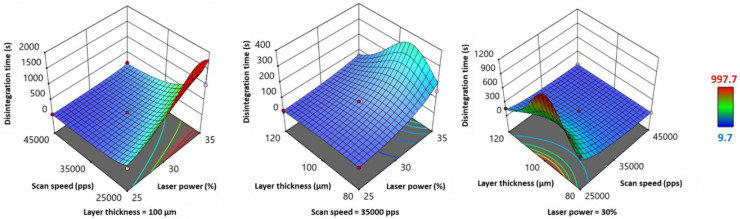
3D response surface plots for disintegration time.

The variations of disintegration time showed similar tendencies to those of weight and hardness (Figure 8). However, the response transform $\frac{1}{\sqrt{Y_{5}-0.5}}$ showed opposite tendencies (Table 6), as it was inversely proportional to the initial Y_5_ response. Therefore, laser power and layer thickness exhibited a significant negative effect, and inversely, scan speed presented a significant positive effect. From Figure 8, it can be observed that Y_5_ values jumped at high *LP* (35%) and low *SS* (25,000 pps), and this interaction effect was significant (Table 6). Regarding the other factors interactions, disintegration time was maximal for the following combinations: high *LP* and *LT* ≈ 105 µm, and low *SS* and *LT* ≈ 100 µm (Figure 8). However, the effect of these two interactions was not significant (Table 6). Furthermore, *LT^2^* showed a significant positive effect on disintegration time (response transform).

**Figure 9:**
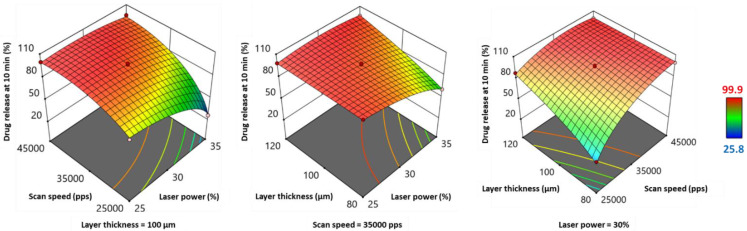
3D response surface plots for percentage of drug release at 10 min.

Concerning the percentage of drug release at 10 min (Y_6_) and its transform Y_6_^2.21^, they displayed opposite variations to weight, hardness, and disintegration time, but similar variations to the response transform $\frac{1}{\sqrt{Y_{5}-0.5}}$ (Table 6 and Figure 9). Indeed, the response transform was significantly positively influenced by scan speed, but laser power and layer thickness presented a significant negative effect (Table 6). All three factors’ interactions exhibited a significant influence on drug release, positive for LP*SS and LP*LT, and negative for SS*LT (Table 6). Figure 9 shows that, while the percentage of drug release at 10 min was usually around 90–100%, it declined at these following parameters combinations: high *LP* and low SS; high *LP* and low *LT*; and low *SS* and low *LT*. Moreover, quadratic terms *LP*^2^ and *SS^2^* demonstrated a significant negative impact on drug release.

The evolution of weight, hardness, disintegration time, and percentage of drug release at 10 min as a function of laser power (*LP*), scan speed (*SS*), and layer thickness (*LT*) is a consequence of variations in energy density (ED). According to the empirical equation, an increase in *LP*, a slow SS, and a low *LT* contribute to an increase in ED. Unlike previously used printers for SLS of SOFs, such as Sintratec^®^ Kit [15], the Sharebot^®^ SnowWhite SLS printer offers the possibility of controlling the power of the CO_2_ laser, thus the amount of energy transmitted by unit time. Furthermore, lowering the scan speed increases the contact time between the powder bed and the laser beam, resulting in high ED. As for the layer thickness, a low value will allow the laser beam to penetrate further, and strongly fuse the particles of two subsequent layers. A low ED would generate less dense SOFs with low weight values. Porous SOFs would break easily, and present low hardness. Porosity is also determinant for disintegration, as pores condition the penetration of the medium. Consequently, porous SOFs would exhibit a low disintegration time. Concerning the drug release, it would be accelerated, as rapid disintegration would permit the polymer to erode rapidly and the drug to dissolve faster. Therefore, a high percentage of drug release would be obtained after 10 min. This explains the results exposed above. For instance, during the printing conditions of Run 14 (*LP* = 25%, *SS* = 25,000 pps and *LT* = 100 µm), the mean values of the dependent variables were: Y_3_ = 143.9 mg, Y_4_ = 44.4 N, Y_5_ = 152 s and Y_6_ = 76.8%. By increasing *LP* (25% to 35%) while maintaining the other independent variables as constant, the weight, hardness, and disintegration time increased significantly, while the percentage of drug release at 10 min declined (Run 7: Y_3_ = 187.2 mg, Y_4_ = 82.6 N, Y_5_ = 997.7 s and Y_6_ = 25.8%). However, by switching the value of *SS* from 25000 to 45000 pps while setting the other parameters as constant, inverse tendencies were observed (Run 11: Y_3_ = 101.7 mg, Y_4_ = 10.6 N, Y_5_ = 9.7 s and Y_6_ = 99.9%). This relationship between the four responses was also demonstrated by the work of Mohamed et al. [31].

Furthermore, low layer thickness (80 µm) increases the energy density, which would result in better densification and reduced porosity. However, higher disintegration time was observed at *LT* = 100 µm, and not at *LT* = 80 µm as expected (Figure 8). This could be explained by the abrasion that occurs at low *LT* (Figure 4) that can affect the internal structure and accelerate the disintegration of the printed SOFs, especially at low *LP* (Run 9) or high *SS* (Run 13).

In addition, layer thickness as a term exhibited a positive effect on weight, hardness, disintegration time, and a negative influence on drug release at 10 min. This does not imply that increasing *LT* augments Y_3_, Y_4_, and Y_5_, and decreases Y_6_, as this would contradict the energy density theory. Models cannot be interpreted by the individual factors alone, since their variations depend on all the terms, especially the significant ones [40]. This explains the interest of using response surface graphs, as they provide a more visual assessment of the evolution of the responses as a function of the factors.

**Figure 10:**
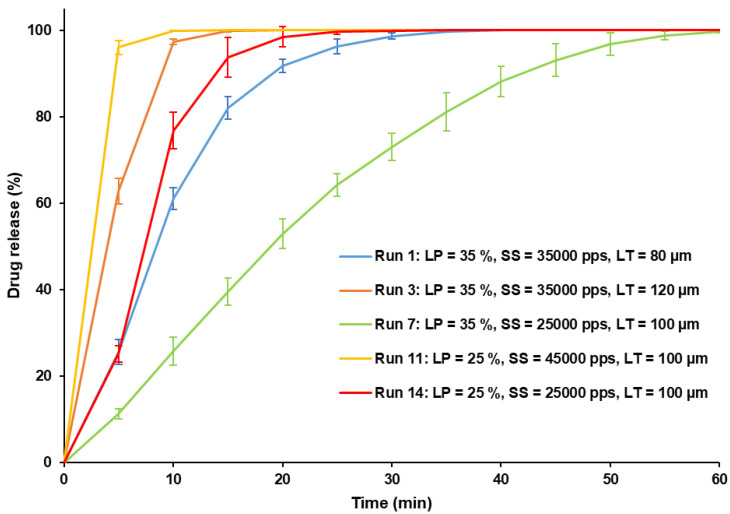
Dissolution profiles of SOFs printed at Runs 1, 3, 7, 11, 14 (N = 3).

Figure 10 displays the dissolution profiles of the printed SOFs under different conditions (Runs 1, 3, 7, 11 and 14). By comparing Run 1 and Run 3, it can be observed that increasing the layer thickness accelerated the drug release. A comparison of Run 14 and Run 11 shows that an increase of the scan speed also promoted quicker dissolution. Finally, a more prolonged drug release was obtained when the laser power was increased (from Run 14 to Run 7). These observations confirm the effect of process parameters on drug release by controlling sintering intensity. It is interesting to note that, without modifying the composition of the formulation and only by changing the settings, SLS allows for a switch from a flash release (100% of drug release within 10 min for Run 11) to a slow release (drug release completed after 1 h for Run 7).

Modeling of dissolution profiles was performed in order to determine which mathematical model best fits the drug release kinetics. Based on the values of RMSE, the Weibull model describes most appropriately the kinetics of drug release for the SOFs printed at each run. This suggests that the drug release kinetics is maintained for a formulation, regardless of the applied SLS parameters. The Equation (6) represents the Weibull function [45]:

(6) $$Q\left(t\right)=100\times \left(1-e^{{\frac{-t}{a}}^{b}}\right)$$

where *Q*(*t*) is the percentage of drug released at time *t*, and *a*, *b* are constants. Table S2 (Supplementary Materials) presents for each printing run the kinetic parameters (*a* and *b*) of the Weibull equation, as well as the corresponding RMSE. For the 15 runs, a value of *b* > 1 was found, indicating a sigmoid curve (Figure 10). According to Papadopoulou et al. [46], when *b* > 1, the mechanism of drug release is complex. Further investigation will need to be conducted in order to elucidate the drug release mechanism, and link it to the porosity of the SOFs.

### 3.3. Physicochemical Characterization of the Printed SOFs

#### 3.3.1. Porosity of the Printed SOFs

**Figure 11:**
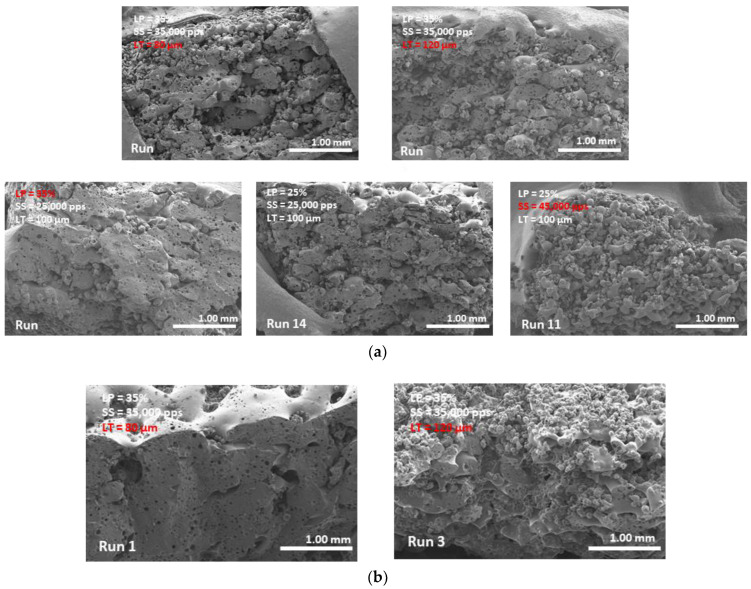
SEM images of SOFs printed at different conditions: (**a**) transversal sections (Runs 1, 3, 7, 11 and 14); (**b**) surface view (Runs 1 and 3) (magnification ×30).

Vertical sections of the printed SOFs in Runs 1, 3, 7, 11, and 14 were visualized using scanning electron microscopy (SEM). SEM images (Figure 11a) illustrate the porosity disparities as a function of the levels of the three process parameters. Taking Run 14 as a reference, it can be observed that SOFs printed with high laser power (*LP*) (Run 7) exhibit a more pronounced fusion of the particles, hence a small number of pores. In the SOFs printed at high scan speed (*SS*) (Run 11), many non-fused particles and pores can be observed (Figure 11a). These observations validate the previously discussed results, as both laser power and scan speed influence the ED, thus the porosity and the dependent variables (weight, hardness, disintegration time, and drug release). Concerning the effect of layer thickness on porosity, this was not accurately assessed by the visualization of the vertical sections on SEM (Figure 11a), as the open pores did not increase considerably from Run 1 *(LT* = 80 µm) to Run 3 *(LT* = 120 µm). However, the SEM surface images (Figure 11b) evidenced indistinct and strongly interpenetrated layers for the SOFs printed during Run 1, and, conversely, distinguishable and weakly consolidated layers for the SOFs printed during Run 3. This confirms the effect of *LT* on bonding two subsequent layers, as previously discussed.

#### 3.3.2. Drug Content in the Printed SOFs

Previously, it was demonstrated that sintering with a CO_2_ laser did not denature paracetamol. However, this statement was only evaluated using a single set of printing parameters (*LP* = 25%, *SS* = 25,000 pps and *LT* = 100 µm) [34]. Thus, API degradation was still a concern in this study, as more extreme printing parameters were applied, especially of high laser power and low scan speed (Run 7). Therefore, UHPLC analysis was performed on the SOFs for each printing run. Results revealed only one chromatographic peak corresponding to paracetamol, with a retention time of 1.26 min, and the average weight-normalized drug content of the SOFs ranging from 98.5% to 102.6% (Supplementary Materials Table S3). This demonstrates that it is possible to vary the properties of the SOFs by varying the process parameters, without affecting the stability of the drug. Nevertheless, this cannot be generalized for other drugs, as they may be more thermolabile than paracetamol.

#### 3.3.3. FTIR Analysis

**Figure 12:**
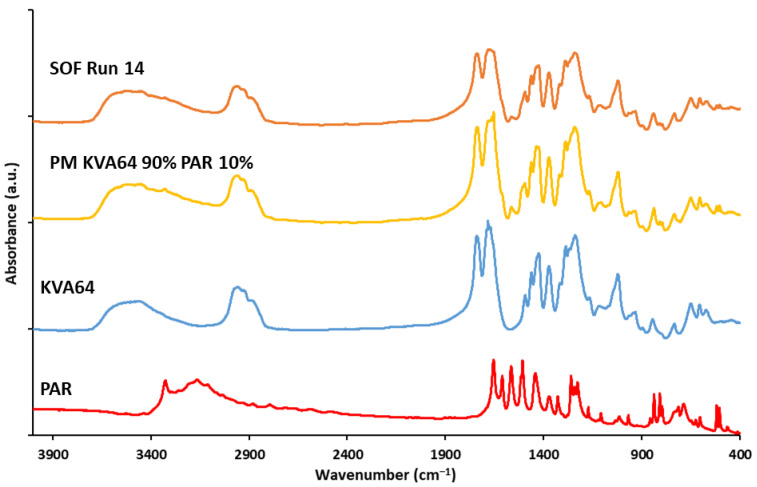
FTIR spectrums of KVA64, PAR, physical mixture, and sintered SOF (Run 14).

The FTIR spectrum of KVA64 (Figure 12) revealed its characteristic peaks at 2946 and 2874 cm^−1^ due to asymmetrical and symmetrical C-H stretching, 1740 cm^−1^ due to C=O stretching of vinyl acetate, and 1683 cm^−1^ due to C=O stretching of the tertiary amide in the pyrrolidone ring. Paracetamol exhibited characteristic peaks at 3318 cm^−1^ due to N-H (amide) stretching, 3153 cm^−1^ due to O-H (phenol) stretching, and 1654 cm^−1^ due to stretching of C=O (amide). In the spectrum of the physical mixture (PM KVA64 90% PAR 10%) (Figure 12), the respective peaks of the two components were present and encompassed. The FTIR spectrum of the printed SOF (Run 14) presented a similar aspect to the PM spectrum, but the intensity of the characteristic peaks of paracetamol was lower, despite analyzing the same weight for both samples. This suggests that the API underwent amorphization in the polymeric matrix. Furthermore, in the printed SOF spectrum, the characteristic peaks of KVA64 at 1740 cm^−1^ and 1683 cm^−1^ were broader and less intense. This could indicate that the corresponding chemical groups, C=O of the vinyl acetate and the C=O of the tertiary amide, may be involved in the formation of hydrogen bonds with H-bonds donors [47], such as paracetamol. This can explain both the amorphization of the drug and its plasticizing effect, as observed in previous work [34].

#### 3.3.4. XRPD Analysis

**Figure 13:**
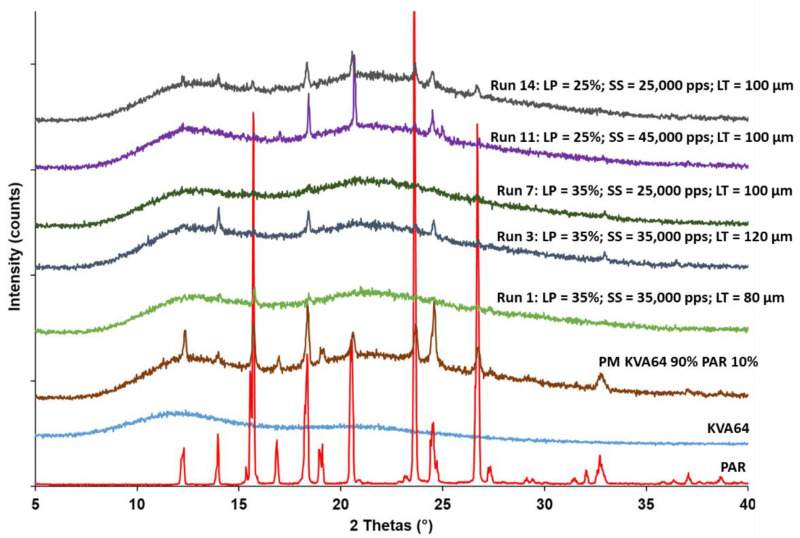
XRPD patterns of KVA64, PAR, physical mixture, and sintered SOFs (Runs 1, 3, 7, 11 and 14).

XRPD patterns (Figure 13) exhibit the amorphous state of KVA64, and the crystalline structure of paracetamol. The crystalline peaks of the API were also found in the physical mixture, but with a lower intensity, as KVA64 was the major component. Furthermore, a decrease in crystallinity was observed in all the SOFs analyzed, suggesting an amorphization of the drug, as demonstrated previously [34]. Comparison between the XRPD patterns of the different runs allowed for the studying of the effect of each process parameter on the solid state of the drug. It should be noted that an increase in laser power (*LP*) (Run 14 vs. Run 7) induced a decrease in crystallinity. Conversely, the crystallinity was higher when the scan speed (*SS*) was increased (Run 14 vs. Run 11). Furthermore, amorphization improved when the layer thickness (*LT*) was reduced (Run 3 vs. Run 1). Overall, an increase in energy density following a variation of the process parameters led to a greater amorphization. This can be explained by a higher fusion of the polymer particles, which promotes the dissolution of the API in the matrix and its amorphization. The effect of energy density on amorphization was also demonstrated in amorphous solid dispersions of lopinavir prepared by SLS at different scan speeds [48].

### 3.4. Optimization Test

Numerical optimization was carried out to determine the optimal values of the critical process parameters (CPPs) to achieve the quality target product profile with the following critical quality attributes (CQAs): maximum printing yield (100%), a target weight of 130 mg, an acceptable hardness (>20 N), a fast disintegration time (<60 s), and a high drug release percentage at 10 min (85–100%). Height was not included among the CQAs, as it is not a critical pharmaceutical aspect. Laser power and scan speed were set within the design range. However, the layer thickness was set as a target value of 100 µm, since it must be a divisor of the design height in order for the number of deposited layers to be a natural number. Only one best-fit solution was generated with the maximum desirability function (Table 7). Desirability is a multiple response method that reflects the desirable ranges for each response. Subsequently, to confirm the validity of the model, an additional experiment was performed to verify the optimized results. The printing conditions and the measured responses of the additional experiment are presented in Table 7. The values of the optimal process parameters were rounded to whole numbers in the confirmation run, as only natural numbers can be input in the printer. All obtained responses were in agreement with the predicted values (within the 95% confidence interval), and matched the CQAs mentioned above.

**Figure 14:**
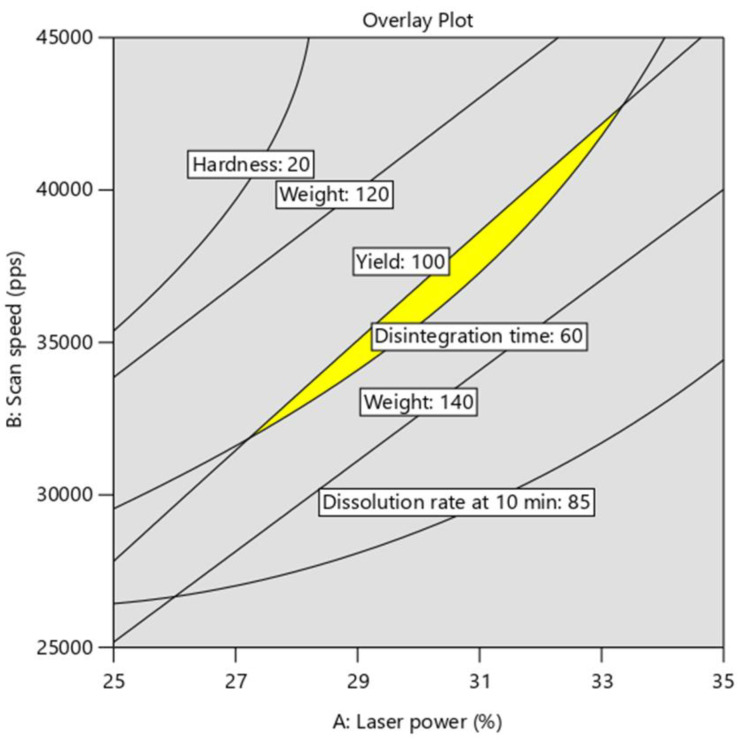
Design space (overlay plot) in function of laser power and scan speed at a constant layer thickness.

In addition to this numerical optimization, a graphical optimization was conducted. This tool can be used to display the design space as recommended by the International Conference on Harmonisation of Technical Requirements for Registration of Pharmaceuticals for Human Use Considerations (ICH) guideline Q8 on pharmaceutical development [28]. The design space displays all combinations of process parameters, satisfying the set of constraints (CQAs) and results from the intersection of the optimal regions for each response. The overlay plot (Figure 14) shows the design space (in yellow) to achieve the desired CQAs as a function of laser power and scan speed, at a constant layer thickness *(LT* = 104.05 µm). It should be noted that the design space is narrow, which indicates the difficulty of obtaining the target properties with various settings. This also explains the unique optimization solution generated by the maximum desirability function (Table 7). Indeed, high printing yield requires high laser power (*LP*) and low scan speed (*SS*) (Figure 3), while fast disintegration time is obtained at low *LP* and high *SS* (Figure 8). This shows that the two CQAs evolve in opposite directions, depending on the two process parameters. Thus, improving printability and producing SOFs with personalized properties (fast drug release and accurate dosage) simultaneously could be troublesome. This explains the interest in using the DoE to define the settings that balance the different CQAs. Additionally, the design space is diagonally shaped, ranging from low levels of *LP* and *SS* to high levels of the two parameters. This implies that at a constant *LT*, both *LP* and *SS* must be proportional in order to maintain an optimal energy density, hence validating the empirical equation.

## 4. Conclusions

This study demonstrates that different processing parameters of selective laser sintering (heating temperature, laser power, scan speed, and layer thickness) influence the printability and properties of solid oral forms printed with copovidone and paracetamol. The first part of this work highlights the importance of an optimal heating temperature to prevent curling of the sintered layers, and to guarantee printability of the formulation. The second part of this work demonstrates that laser power, scan speed, and layer thickness are critical process parameters through a design of experiments, and have a significant effect (*p* < 0.05) on the critical quality attributes of the solid oral forms. Printing yield was negatively affected by low laser power, high scan speed, and low layer thickness as a consequence of the curling phenomenon. Weight, hardness, and disintegration time were proportional to laser power, and inversely proportional to scan speed and layer thickness, whilst the percentage of drug release at 10 min showed opposite variations. The evolution of these four responses was the consequence of the variations in energy density and porosity as a function of the three process parameters. Lastly, an optimization was carried out to determine the optimal process parameters and design space for the defined critical quality attributes. The narrowness of the design space shows that it is challenging to maximize printability and obtain solid oral forms with the targeted properties.

In conclusion, Quality by Design approaches, such as the one presented in this work, provide a better insight into how process parameters affect printability, and can also be used to adapt the properties of printed solid oral forms. The comprehension of this technology is paramount for its implementation at a clinical level, and for achieving personalized medicine through 3D printing.

## Figures and Tables

**Table 1:** Process parameters for each design point.

Run	LP (%)	SS (pps)	LT (µm)
**1**	35	35,000	80
**2**	30	25,000	80
**3**	35	35,000	120
**4**	25	35,000	120
**5**	35	45,000	100
**6**	30	45,000	120
**7**	35	25,000	100
**8**	30	25,000	120
**9**	25	35,000	80
**10**	30	35,000	100
**11**	25	45,000	100
**12**	30	35,000	100
**13**	30	45,000	80
**14**	25	25,000	100
**15**	30	35,000	100

**Table 2:** Printing yield for the powder mixture KVA64 90% / PAR 10% at five different temperatures.

HT (°C)	90	95	100	105	110
**Yield (%)**	11.1	38.9	100	77.8	47.2

HT: heating temperature.

**Table 3:** Properties of the SOFs printed at three different temperatures.

HT (°C)	95	100	105
**Height (mm)**	3.00 ± 0.06	3.07 ± 0.04	3.56 ± 0.13
**Weight (mg)**	135.2 ± 2.7	136.2 ± 1.7	142.3 ± 2.6
**Hardness (N)**	35.0 ± 2.7	36.5 ± 2.8	36.3 ± 2.9
**Disintegration time (s)**	72.3 ± 4.2	79.3 ± 5.5	88.0 ± 4.6
**Drug release at 10 min (%)**	96.3 ± 4.1	95.7 ± 1.2	89.9 ± 4.0

**Table 4:** Measured responses for each design point.

Run	Yield (%)	Height (mm)	Weight (mg)	Hardness (N)	Disintegration Time (s)	Drug Release at 10 min (%)
**1**	94.4	3.96 ± 0.06	180.4 ± 4.7	53.4 ± 3.3	146 ± 8.9	61.2 ± 2.5
**2**	97.2	3.67 ± 0.09	180.6 ± 2.3	83.4 ± 3.2	402.3 ± 9.6	39.7 ± 3.5
**3**	100.0	2.92 ± 0.08	125.1 ± 2.0	25.7 ± 2.7	61.7 ± 5.7	97.4 ± 0.6
**4**	97.2	3.07 ± 0.06	101.9 ± 2.9	9.2 ± 1.4	16.3 ± 4.7	98.6 ± 0.8
**5**	100.0	3.01 ± 0.03	132.9 ± 1.0	44.9 ± 2.2	133.0 ± 6.0	96.4 ± 2.5
**6**	88.9	2.93 ± 0.05	98.2 ± 2.4	10.9 ± 0.8	12.3 ± 2.5	99.8 ± 0.2
**7**	100.0	3.33 ± 0.02	187.2 ± 2.5	82.6 ± 3.9	997.7 ± 20.3	25.8 ± 3.2
**8**	100.0	3.03 ± 0.06	138.0 ± 1.9	33.2 ± 3.2	166.0 ± 4.6	87.9 ± 3.8
**9**	55.6	3.49 ± 0.04	125.0 ± 3.2	19.7 ± 2.5	26.7 ± 5.7	99.8 ± 0.2
**10**	100.0	3.09 ± 0.05	137.0 ± 2.1	37.6 ± 3.6	71.7 ± 7.5	94.2 ± 2.6
**11**	75.0	3.04 ± 0.03	101.7 ± 1.4	10.6 ± 1.0	9.7 ± 0.6	99.9 ± 0.1
**12**	100.0	3.15 ± 0.05	141.2 ± 1.6	39.8 ± 1.3	67.3 ± 3.2	97.1 ± 2.4
**13**	63.9	3.49 ± 0.04	121.5 ± 1.3	12.2 ± 1.4	23.7 ± 4.5	99.5 ± 0.4
**14**	100.0	3.14 ± 0.06	143.9 ± 1.1	44.4 ± 2.8	152.0 ± 10.8	76.8 ± 4.3
**15**	100.0	3.07 ± 0.04	136.2 ± 1.7	36.5 ± 2.8	79.3 ± 5.5	95.7 ± 1.2

**Table 5:** ANOVA analysis of the models for the different responses.

Response	Yield		Height		Weight		Hardness		Disintegration Time *		Drug Release at 10 min *	
**Source**	F-value	*p*-value	F-value	*p*-value	F-value	*p*-value	F-value	*p*-value	F-value	*p*-value	F-value	*p*-value
**Model**	12.28	0.0065	40.27	0.0004	165.41	<0.0001	28.49	0.0009	61.32	0.0001	93.99	<0.0001
**A-Laser power**	21.49	0.0057	8.22	0.0351	402.04	<0.0001	62.80	0.0005	171.48	<0.0001	113.28	0.0001
**B-Scan speed**	23.32	0.0048	18.29	0.0079	653.52	<0.0001	113.63	0.0001	279.24	<0.0001	410.63	<0.0001
**C-Layer thickness**	27.20	0.0034	255.60	<0.0001	357.19	<0.0001	33.71	0.0021	27.42	0.0034	102.40	0.0002
**AB**	6.04	0.0573	3.71	0.1122	5.00	0.0757	0.12	0.7427	46.04	0.0011	26.08	0.0037
**AC**	12.61	0.0164	27.72	0.0033	35.45	0.0019	2.47	0.1771	0.14	0.7194	56.61	0.0007
**BC**	4.78	0.0806	0.38	0.5665	12.72	0.0161	19.96	0.0066	3.72	0.1117	53.55	0.0007
**A^2^**	1.72	0.2464	2.99	0.1442	0.43	0.543	0.00	0.9632	2.30	0.1896	16.88	0.0093
**B^2^**	1.10	0.3419	0.82	0.4066	2.85	0.1524	7.48	0.0411	0.21	0.6658	70.84	0.0004
**C^2^**	13.50	0.0144	44.75	0.0011	17.97	0.0082	14.47	0.0126	22.53	0.0051	0.06	0.8203
**Lack of Fit**	/		2.42	0.3054	1.05	0.5217	17.59	0.0543	13.58	0.0694	2.50	0.2986
**Std. Dev.**	5.08		0.06		2.70		5.48		0.01		1108.57	
**Mean**	91.48		3.23		136.71		36.27		0.14		19919.03	
**C.V. %**	5.56		1.82		1.98		15.10		9.89		5.57	
**R^2^**	0.9567		0.9864		0.9967		0.9809		0.9910		0.9941	
**Adjusted R^2^**	0.8788		0.9619		0.9906		0.9464		0.9749		0.9835	
**Predicted R^2^**	0.3074		0.8226		0.9643		0.7035		0.8621		0.9230	
**Adeq Precision**	10.7064		21.4673		41.3073		17.4107		25.8124		27.8508	

*: response transform.

**Table 6:** Quadratic models generated for the different responses.

Response	Equation
**Yield**	Y_1_ = **− 410.938 + 14.653 A − 0.005 B + 7.066 C** + 1.250 × 10^−4^ AB **− 0.090 AC** + 2.778 × 10^−5^ BC − 0.139 A^2^ − 2.778 × 10^−8^ B^2^ **− 0.024 C^2^**
**Height**	Y_2_ = **5.881 + 0.079 A + 3.539 × 10^−5^ B − 0.075 C** − 1.130 × 10^−6^ AB **− 0.002 AC** + 9.000 × 10^−8^ BC + 0.002 A^2^ − 2.767 × 10^−10^ B^2^ **+ 5.110 × 10^−4^ C^2^**
**Weight**	Y_3_ = **− 108.419 + 11.789 A − 0.004 B + 3.648 C** − 6.000 × 10^−5^ AB **− 0.080 AC + 2.400 × 10^−5^ BC** + 0.037 A^2^ + 2.373 × 10^−8^ B^2^ **− 0.015 C^2^**
**Hardness**	Y_4_ = **− 41.066 + 8.365 A − 0.013 B + 4.007 C** − 1.900 × 10^−5^ AB − 0.043 AC **+ 6.100 × 10^−5^ BC** − 0.006 A^2^ **+ 7.791 × 10^−8^ B^2^ − 0.027 C^2^**
**Disintegration time**	$\frac{1}{\sqrt{Y_{5}-0.5}}$ = **0.559 − 0.004 A + 2.800 × 10^−5^ B − 0.018 C − 9.692 × 10^−7^ AB** − 2.700 × 10^−5^ AC + 6.866 × 10^−8^ BC + 4.510 × 10^−4^ A^2^ + 3.409 × 10^−11^ B^2^ **+ 8.800 × 10^−5^ C^2^**
**Percentage of drug release at 10 min**	Y_6_^2.21^ = **− 33432.544 − 1298.081 A + 4.523 B − 273.923 C + 0.057 AB + 41.703 AC − 0.020 BC − 94.803 A^2^ − 4.900 × 10^−5^ B^2^** − 0.345 C^2^

A: laser power; B: scan speed; C: layer thickness; Y_1_: yield; Y_2_: height; Y_3_: weight; Y_4_: hardness; Y_5_: disintegration time; Y_6_: percentage of drug release at 10 min.

**Table 7:** Optimization results and the measured responses for the confirmation test.

Settings	LP (%)	SS (pps)	LT (µm)	Y1 (%)	Y2 (mm)	Y3 (mg)	Y4 (N)	Y5 (s)	Y6 (%)	Desirability
**Solution**	30.67	37,447.45	104.05	100.5	3.03	130.9	33.2	56.4	98.9	0.218
**Confirmation**	31	37447	100	100	3.03 ± 0.03	133.1 ± 1.7	34.0 ± 3.5	53.3 ± 3.2	95.3 ± 0.7	/

LP: laser power; SS: scan speed; LT: layer thickness; Y_1_: yield; Y_2_: height; Y_3_: weight; Y_4_: hardness; Y_5_: disintegration time; Y_6_: percentage of drug release at 10 min.

## Data Availability

Data is contained within the article or Supplementary Materials.

## Supplementary Materials

The following are available online at https://www.mdpi.com/article/10.3390/pharmaceutics13101701/s1, Figure S1. Aspect of the powder bed after SLS printing: (a) presence of curling, (b) absence of curling. Figure S2. Dissolution profiles of SOFs printed at different temperatures (95, 100, 105 °C). Figure S3. Residuals vs. run plots: (a) yield, (b) height, (c) weight, (d) hardness, (e) disintegration time (response transform), (f) percentage of drug release at 10 min (response transform). Figure S4. Plots of predicted vs. actual responses: (a) yield, (b) height, (c) weight, (d) hardness, (e) disintegration time (response transform), (f) percentage of drug release at 10 min (response transform). Table S1. Average thickness of the sintered layer for each printing run. Table S2. Kinetic parameters (a and b) of Weibull model fitted to the dissolution profiles of SOFs printed at each run and the corresponding root mean square error (RMSE). Table S3. Drug content of the SOFs printed at each run.
