# Supplementary material for: A QbD Approach for Evaluating the Effect of Selective Laser Sintering Parameters on Printability and Properties of Solid Oral Forms
Source: Pharmaceutics. 2021 Oct 15;13(10):1701. doi: 10.3390/pharmaceutics13101701 (PMC8539463; doi:10.3390/pharmaceutics13101701)
Supplement: Supplementary file 1 [file pharmaceutics-13-01701-s001.zip › pharmaceutics-1395488-supplementary.pdf]

pharmaceutics

Supplementary Materials: A QbD Approach for Evaluating the Effect of Selective Laser Sintering Parameters on Printability and Properties of Solid Oral Forms

Yanis A Gueche, Noelia M Sanchez-Ballester, Bernard Bataille, Adrien Aubert, Jean-Christophe Rossi and Ian Soulairol

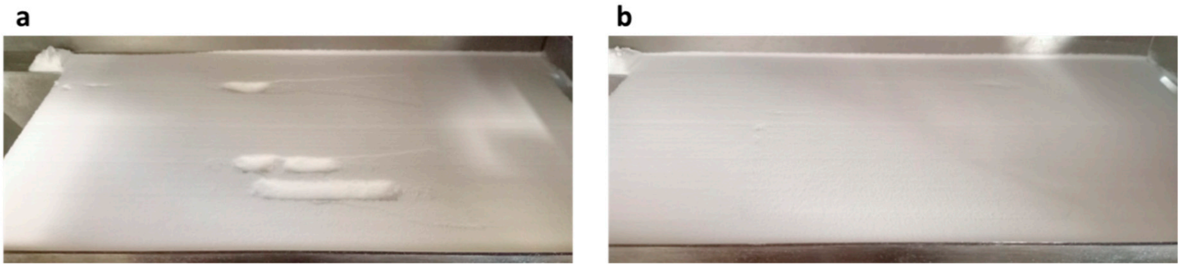

Figure S1. Aspect of the powder bed after SLS printing: (**a**) presence of curling, (**b**) absence of curling.

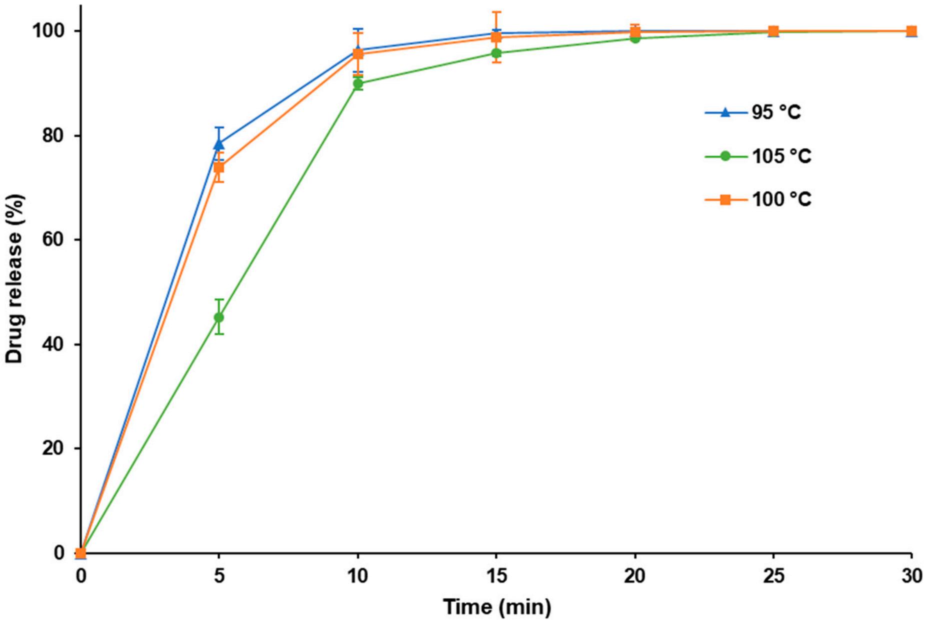

Figure S2. Dissolution profiles of SOFs printed at different temperatures (95, 100, 105 °C).

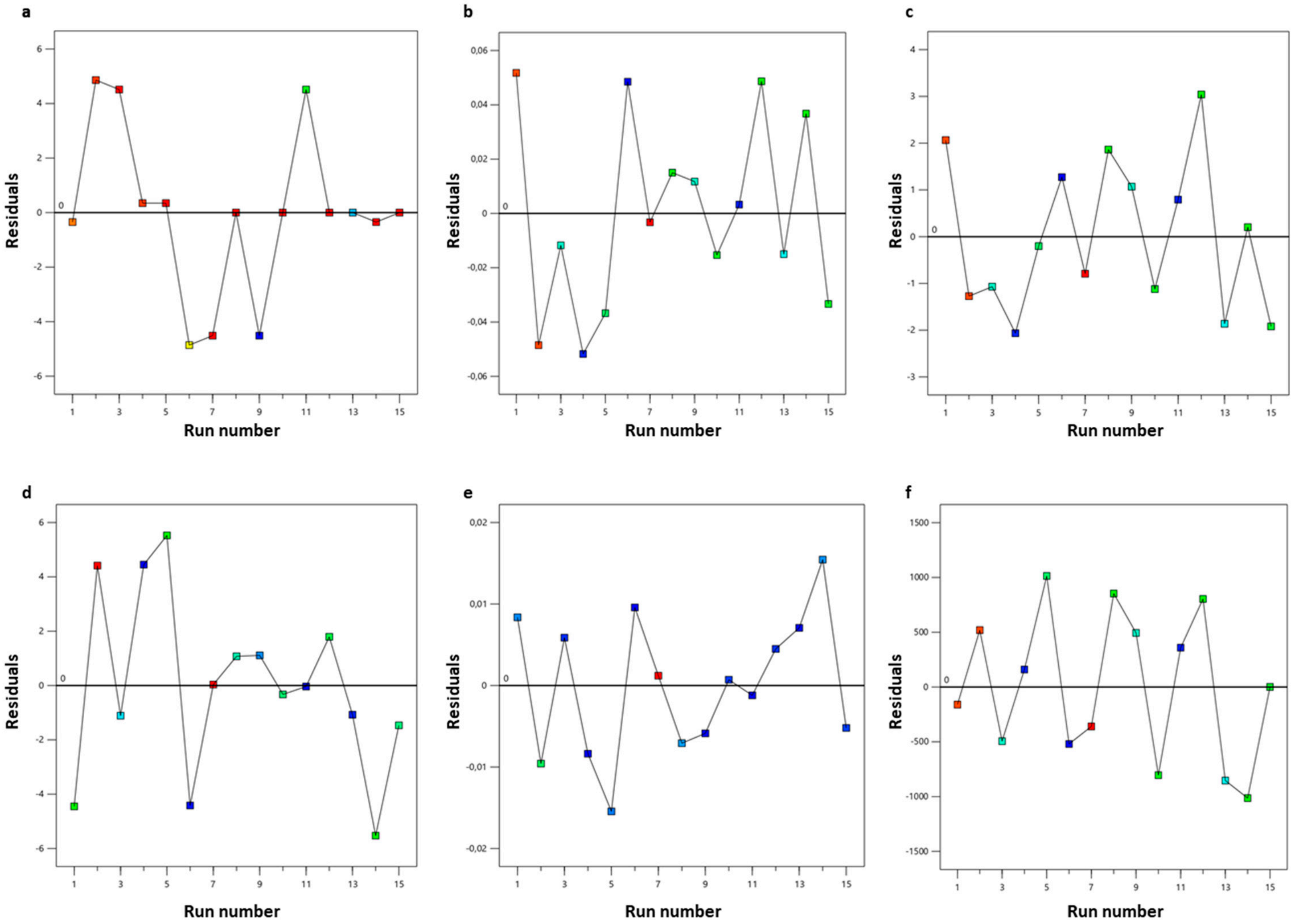

Figure S3. Residuals *vs.* run plots: (**a**) yield, (**b**) height, (**c**) weight, (**d**) hardness, (**e**) disintegration time (response transform), (**f**) percentage of drug release at 10 min (response transform).

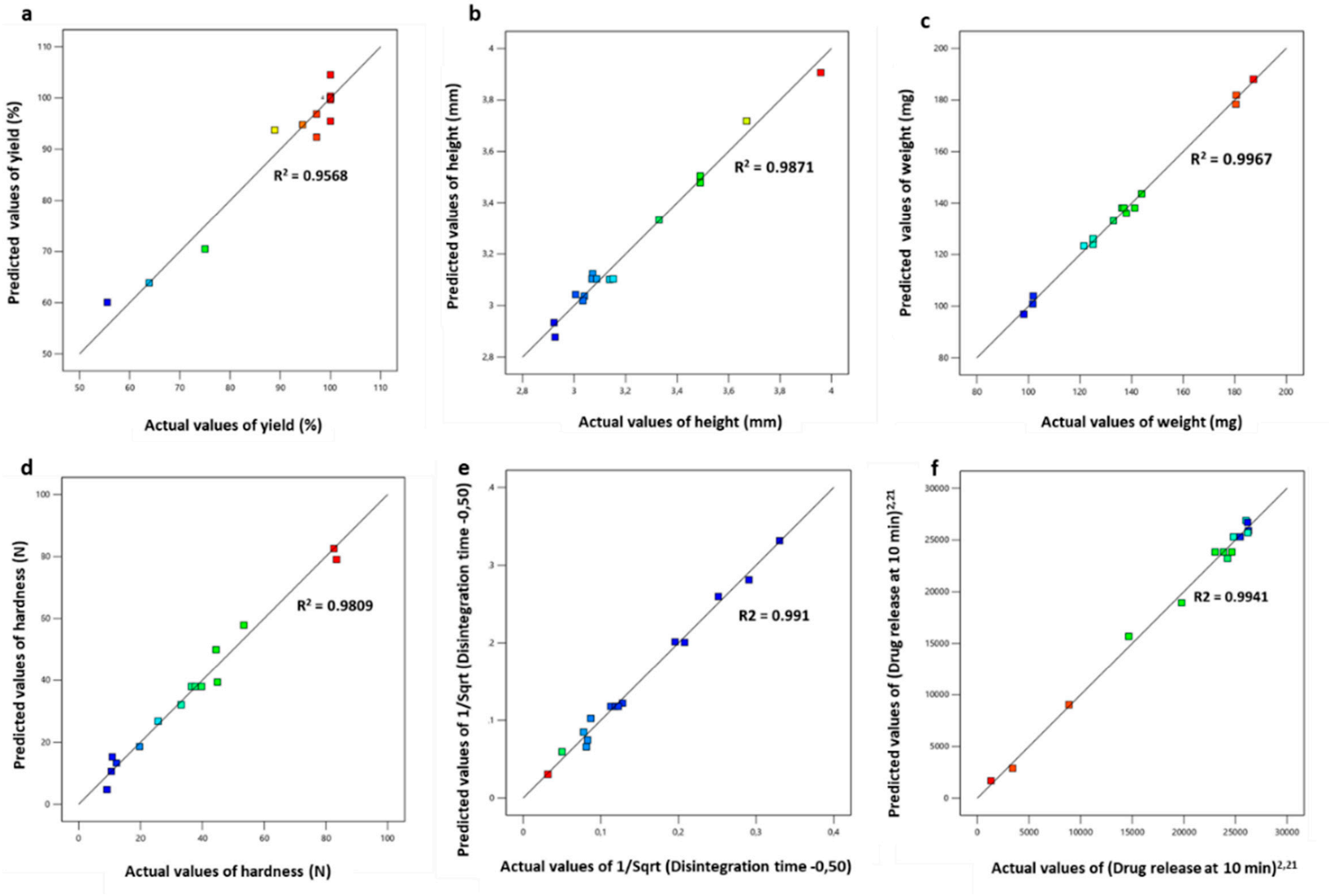

Figure S4. Plots of predicted *vs.* actual responses: (**a**) yield, (**b**) height, (**c**) weight, (**d**) hardness, (**e**) disintegration time (response transform), (**f**) percentage of drug release at 10 min (response transform).

Table S1. Average thickness of the sintered layer for each printing run.

Run	Height of SOF (mm)	LT (μm)	ASLT (μm)
**1**	3.96	80	131.9
**2**	3.67	80	122.3
**3**	2.92	120	146.1
**4**	3.07	120	153.6
**5**	3.01	100	125.3
**6**	2.93	120	146.3
**7**	3.33	100	138.8
**8**	3.03	120	151.7
**9**	3.49	80	116.3
**10**	3.09	100	128.7
**11**	3.04	100	126.7
**12**	3.15	100	131.3
**13**	3.49	80	116.3
**14**	3.14	100	130.8
**15**	3.07	100	127.9

Table S2. Kinetic parameters (**a** and **b**) of Weibull model fitted to the dissolution profiles of SOFs printed at each run and the corresponding root mean square error (RMSE).

Run	a	b	RMSE
**1**	6.1080	1.1321	0.6832
**2**	1.0068	1.1126	1.7795
**3**	2.8423	1.1556	0.0405
**4**	2.6304	1.1557	0.0546
**5**	2.8369	1.1557	0.0103
**6**	2.2815	1.1585	0.0045
**7**	1.9790	1.0805	1.4024
**8**	3.5157	1.1509	0.2963
**9**	2.2422	1.1586	0.0083
**10**	3.1812	1.1517	0.0252
**11**	2.2709	1.1587	0.0002
**12**	2.8103	1.2477	0.0948
**13**	2.5282	1.1565	0.0062
**14**	4.6830	1.1421	0.9509
**15**	3.1848	1.1515	0.1636

Table S3. Drug content of the SOFs printed at each run.

Run	Weight-normalized Drug Content (%)
**1**	102.6 ± 5.4
**2**	100.8 ± 1.4
**3**	99.9 ± 1.3
**4**	99.8 ± 2.3
**5**	102.6 ± 2.8
**6**	98.5 ± 1.0
**7**	101.7 ± 1.8
**8**	100.6 ± 2.1
**9**	99.9 ± 7.6
**10**	99.7 ± 1.8
**11**	99.5 ± 4.3
**12**	100.5 ± 4.3
**13**	99.7 ± 0.3
**14**	101.6 ± 2.2
**15**	99.9 ± 1.8
